# Improved reproducibility of LV volumetry and infarct size measurement using a standardized evaluation protocol for cardiac magnetic resonance imaging

**DOI:** 10.1186/1532-429X-13-S1-P160

**Published:** 2011-02-02

**Authors:** Gunnar K Lund, Michael Groth, Kai Müllerleile, Thorsten Klinik, Soraya Halaj, Gregor Folwarski, Dennis Säring, Gerhard Adam

**Affiliations:** 1 Radiology, Hamburg, Germany; 2 Heart Center, Hamburg, Germany; 3 Medizinische Informatik, Hamburg, Germany

## Purpose

LV volumetry and infarct size measurement using cardiac magnetic resonance imaging (MRI) is the reference standard to non-invasively measure and to quantify cardiac volumes, function and infarct size. We have observed a high variability of cardiac MRI measurements, if inexperienced observers evaluated the MRI data. The purpose of the study was to analyze the reproducibility of cardiac MRI measurements of cardiac volumes, function, LV mass and infarct size before and after training including the use of a standardized evaluation protocol.

## Materials and methods

Cardiac MRI data of 10 patients with myocardial infarction were analyzed by 2 experienced and 4 inexperienced observers in respect to end-diastolic volume (EDV), end-systolic volume (ESV), ejection fraction (EF), LV mass and infarct size. All planimetric measurements were performed on short axis slices according to the Simpson´s method using a dedicated software (HeAT). The mean of the two experienced observers served as the reference standard. Subsequently, the inexperienced observers were trained which included the explanation of accepted rules to trace the end- and epicardial contours of the myocardium and to delineate the trabeculae and the papillary muscles. Thereafter, the experienced and the trained observers analyzed the MRI data of another 10 patients.

## Results

Before training the mean variability of the inexperienced observers was 3.6 ±9.6% for EDV, 11.9 ±17.5% for ESV, -5.6 ±8.1% for EF, 10.7 ±11.0% for LV mass and 26.1 ±28.3% for infarct size. After training and adherence to a standard evaluation protocol, the variability significantly improved to -1.6 ±7.3% for EDV (P<0.01), to 0.6 ±9.1 for ESV (P<0.001), to -2.0 ±6.5% for EF (P<0.05), and to 5.5 ±15.7 % for infarct size (P<0.0001). No improvement was found for LV mass which was 9.4 ±6.4% after training (P=ns).

## Conclusion

Training and adherence to a standardized evaluation protocol significantly reduced the variability of EDV, ESV, EF and infarct size.

